# A macrocyclic peptide inhibitor traps MRP1 in a catalytically incompetent conformation

**DOI:** 10.1073/pnas.2220012120

**Published:** 2023-03-09

**Authors:** Harlan L. Pietz, Ata Abbas, Zachary Lee Johnson, Michael L. Oldham, Hiroaki Suga, Jue Chen

**Affiliations:** ^a^Laboratory of Membrane Biology and Biophysics, The Rockefeller University, New York, NY 10065; ^b^Department of Chemistry, School of Science, The University of Tokyo, Tokyo 113-0033, Japan; ^c^HHMI, New York, NY 10065

**Keywords:** ABC transporter, multidrug resistance protein 1, cyclic peptide, inhibition

## Abstract

Adenosine triphosphate-binding cassette (ABC) transporters, such as multidrug resistance protein 1 (MRP1), protect against cellular toxicity by exporting xenobiotic compounds across the plasma membrane. However, constitutive MRP1 function hinders drug delivery across the blood–brain barrier, and MRP1 overexpression in certain cancers leads to acquired multidrug resistance and chemotherapy failure. Small-molecule inhibitors have the potential to block substrate transport, but few show specificity for MRP1. Here we identify a macrocyclic peptide, named CPI1, which inhibits MRP1 with nanomolar potency but shows minimal inhibition of a related multidrug transporter P-glycoprotein. A cryoelectron microscopy (cryo-EM) structure at 3.27 Å resolution shows that CPI1 binds MRP1 at the same location as the physiological substrate leukotriene C4 (LTC_4_). Residues that interact with both ligands contain large, flexible sidechains that can form a variety of interactions, revealing how MRP1 recognizes multiple structurally unrelated molecules. CPI1 binding prevents the conformational changes necessary for adenosine triphosphate (ATP) hydrolysis and substrate transport, suggesting it may have potential as a therapeutic candidate.

## Significance

We have used an established in vitro cyclic peptide selection strategy to generate a novel inhibitor of multidrug resistance protein 1 (MRP1), an ABC transporter implicated in cancer drug resistance. Multiple small-molecule MRP1 inhibitors have been described in the literature, many of which were first discovered to be inhibitors of P-glycoprotein (Pgp). The cyclic peptide we have generated, CPI1, exhibits robust inhibition of MRP1 but minimal inhibition of Pgp. The structure of MRP1 in complex with CPI1—the first of MRP1 bound to an inhibitor of any sort—reveals the mechanism of MRP1 inhibition and illustrates how MRP1 may bind a diverse range of ligands.

Multidrug resistance protein 1 (MRP1) is an ATP-binding cassette (ABC) transporter widely expressed in the lung, kidney, placenta, heart, colon, brain, small intestine, and particularly the blood–brain and blood–testis barriers (1–5). By transporting physiological substrates such as leukotriene C_4_ (LTC_4_), MRP1 serves to regulate redox homeostasis, steroid metabolism, inflammation, and hormone secretion (6, 7). Its ability to export xenobiotic compounds out of cells protects tissues from toxicity (8), but this function accordingly reduces the efficacy of antidepressants, antivirals, and antibiotics (6, 9–11). MRP1 overexpression is associated with resistance to chemotherapy and poor clinical outcomes for cancers such as acute myeloblastic and lymphoblastic leukemia, non-small cell lung cancer, prostate cancer, breast cancer, and particularly neuroblastoma (12–18). MRP1-mediated multidrug resistance in neuroblastoma likely occurs because three first-line chemotherapeutic treatments—doxorubicin, etoposide, and vincristine (19)—are all MRP1 substrates. For these reasons, MRP1 inhibitors may assist drug delivery across biological barriers and overcome drug resistance during chemotherapy.

MRP1 is a single polypeptide with five structural domains: an N-terminal transmembrane domain (TMD0) plus a lasso motif (L0), and the canonical ABC transporter architecture of two transmembrane domains (TMD1 and TMD2) and two nucleotide-binding domains (NBD1 and NBD2) (20). Structures of bovine MRP1 (bMRP1) have been determined by cryo-EM in four functional states: an apo (inward-facing) form, an LTC_4_-bound (inward-facing) form, an ATP-bound (outward-facing) form stabilized by an ATP hydrolysis-defective mutation, and a post-hydrolytic (outward-facing) form (20–22). Together, these structures have revealed that MRP1 uses the well-described alternating access mechanism, in which ATP-dependent conformational changes expose the substrate-binding site to alternate sides of the membrane and thereby drive substrate translocation from one side to the other.

Abrogation of cancer drug resistance by pharmacological inhibition of MRP1 has long been pursued (10). An early study revealed that MRP1 knockout mice are viable and fertile, but hypersensitive to the anticancer drug etoposide, suggesting that MRP1 inhibition is a feasible option to increase the efficacy of chemotherapy (23). However, the majority of available MRP1 inhibitors are repurposed from other drug discovery efforts and thus have inherent off-target issues (24, 25). For example, the small-molecule inhibitors LY475776 and Reversan also inhibit P-glycoprotein (Pgp), a homologous drug transporter with a broad substrate spectrum (26–28).

Macrocyclic peptides have the notable advantage over small molecules of a much larger binding interface, which may result in greater affinity and selectivity (29). Furthermore, cyclization limits conformational flexibility and increases resistance to cellular proteases (30). Indeed, of the ~60 peptide therapeutics that have been approved for clinical use (31), more than 40 are cyclic (30). An effective method to identify macrocyclic ligands for a specific protein is the random nonstandard peptides integrated discovery (RaPID) system, which combines messenger RNA display and flexible in vitro translation (FIT) strategies to construct a highly diverse peptide library (32). This method has been used to identify high-affinity ligands for several clinically relevant drug targets (33–40) as well as a bacterial ABC transporter TmrAB (41).

In this study, we used the RaPID system to identify a macrocyclic peptide that inhibits MRP1 with an inhibition constant (K_i_) of 100 nM. A cryo-EM structure of the peptide-MRP1 complex reveals that the peptide binds to the substrate-binding site and prevents conformational changes essential for ATP hydrolysis and substrate translocation. Although the potential of this peptide as a drug candidate awaits future studies, it nevertheless represents a valuable tool to investigate the molecular mechanism of MRP1 substrate transport.

## Results

### CPI1 Is a Specific Inhibitor of MRP1 Over Pgp.

**Fig. 1.**
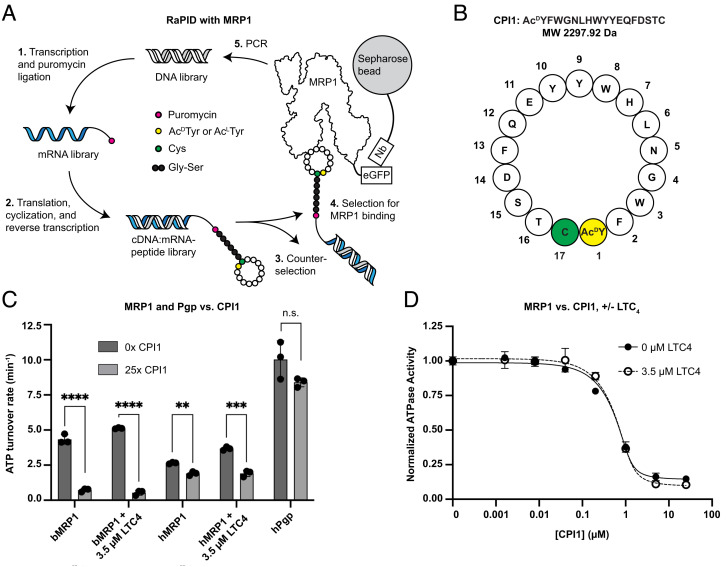
Identification of an MRP1-specific inhibitor. (*A*) Selection strategy for novel cyclic peptide MRP1 ligands. eGFP: enhanced green fluorescent protein; Nb: anti-eGFP nanobody. (*B*) CPI1 composition. The first residue in the sequence (Ac^D^Y1, yellow) is covalently bonded to the 17th residue (C17, green) via a thioether linkage. (*C*) ATPase activity of detergent-solubilized hMRP1 (1 µM), bMRP1 (1 µM), and Pgp (0.5 µM) at 4 mM ATP in the presence or absence of CPI1 (25-fold molar excess). All values reported as the mean ± standard error (SE) of three separate measurements. The statistical significance was calculated using unpaired, parametric, two-sided *t* tests. Labels: not significant (n.s.), *P* < 0.05 (*), *P* < 0.01 (**), *P* < 0.001 (***) *P* < 0.0001 (****). *P*-values: bMRP1, 0.000065; bMRP1 + 3.5 µM LTC4, 0.000002; hMRP1, 0.001153; hMRP1 + LTC4, 0.000277; hPgp, 0.107. (*D*) Dose–response curves of the basal and LTC_4_-stimulated ATPase activity of bMRP1. Rates of ATP hydrolysis were normalized to maximal activity. Data were fit to a modified version of the quadratic binding equation to account for free ligand depletion (*Materials and Methods*).

To identify cyclic peptides that bind to MRP1, we constructed a peptide library as previously described (Fig. 1*A*) (32). Briefly, a collection of semirandomized DNA sequences was generated, each consisting of a start codon, a stretch of 4 to 15 NNK nucleotides (N = A, G, C, or T; K = T or G), a Cys codon, and finally three Gly-Ser codon pairs. Each sequence in the library was subjected to in vitro transcription, 3′ puromycin ligation, and FIT using a genetically reprogrammed system in which the initial methionine of each peptide is replaced with either a D- or L-enantiomer of *N-*chloroacylated tyrosine (ClAc^L^Y or ClAc^D^Y). This leading N-chloroacylated tyrosine spontaneously reacts with the downstream cysteine to form a thioether-linked macrocyclic structure. Following translation of the Gly-Ser codon series, each cyclic peptide was ligated to its cognate mRNA via the 3′ puromycin moiety. Reverse transcription of the cognate mRNA yielded a library of cDNA:mRNA-cyclic peptide molecules. Two cyclic peptide libraries—one with leading ClAc^L^Y and one with leading ClAc^D^Y amino acids—were subjected to an affinity screen against full-length human MRP1 (hMRP1) purified in digitonin and immobilized to nanobody-conjugated Sepharose beads via a C-terminal Green Fluorescent Protein (GFP) tag. After counterselection against the Sepharose-GFP nanobody beads, cDNA molecules corresponding to hMRP1-bound ligands were eluted, amplified by PCR, and identified by sequencing.

This protocol yielded 10 candidates—five from the ClAc^L^Y library and five from the ClAc^D^Y library—which were tested for their functional effects. A peptide from the ClAc^D^Y library, which we named cyclic peptide inhibitor 1 (CPI1), exhibited robust inhibition of hMRP1 ATPase activity and was selected for further characterization. CPI1 comprises 17 residues: an N-acylated D-tyrosine (Ac^D^Y) followed by 15 proteinogenic amino acids (Phe-Trp-Gly-Asn-Leu-His-Trp-Tyr-Tyr-Glu-Gln-Phe-Asp-Ser-Thr) and a cysteine that forms the thioether linkage with Ac^D^Y (Fig. 1*B*). Although hMRP1 was used for ligand selection, CPI1 inhibited the ATPase activity of both human and bovine MRP1 (Fig. 1*C*), which share 91% sequence identity and are functionally redundant (42). Because bMRP1 is biochemically more stable than hMRP1 and its high-resolution structures are available, we sought to further characterize CPI1 using bMRP1. The K_i_ values of CPI1 against bMRP1 were determined to be 70 ± 29 nM and 95 ± 46 nM in the absence and presence of 3.5 µM LTC_4_, respectively (Fig. 1*D*). In contrast to multiple previously reported small-molecule inhibitors of MRP1 (26–28), CPI1 resulted in only modest inhibition of Pgp when applied at a 25-fold molar excess (Fig. 1*C*). Thus, CPI1 is a highly specific and effective inhibitor of MRP1.

### CPI1 Binds to the Same Binding Site as LTC_4_.

**Fig. 2.**
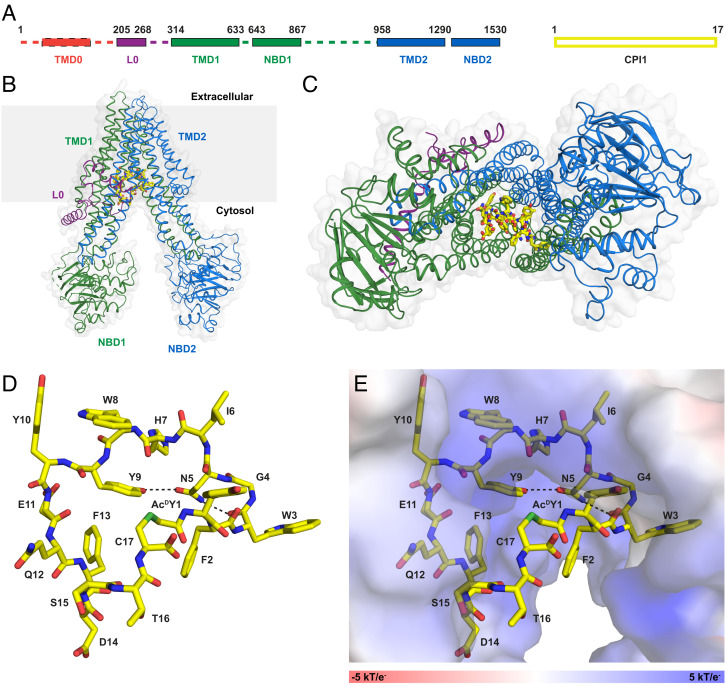
Cryo-EM structure of bMRP1 in complex with CPI1. (*A*) Schematic drawing of bMRP1 and CPI1. Sequences and domains (TMD0) denoted by dashed lines were not resolved in the cryo-EM reconstruction. (*B*) The overall architecture of the complex. CPI1 is shown in yellow stick representation. The membrane is indicated by a grey box. (*C*) CPI1 binds at the interface of the TMDs. (*D*), Molecular structure of CPI1. Two intramolecular hydrogen bonds (Y9-N5, and N5-W3) are indicated by dashed lines. (*E*) CPI1 nearly fills the TM cavity, with the S15-C17 region largely solvent-exposed. MRP1 is shown as an electrostatic surface. CPI1 is colored by heteroatom (C yellow, O red, N blue) with the thioether bond shown in green.

A cryo-EM structure of the CPI1–bMRP1 complex in the absence of substrate and ATP was determined to an overall resolution of 3.27 Å (Fig. 2 and *SI Appendix*, Figs. S1 and S2 and Table S1). The cryo-EM map shows well-defined density for the peptide inhibitor, permitting unambiguous residue assignment (*SI Appendix*, Fig. S2). The final atomic model (8F4B/EMD-28854) included all 17 residues of CPI1 as well as L0, TMD1, NBD1, TMD2, and NBD2 of MRP1 (Fig. 2*A*). TMD0 was too flexible to be resolved and not included in the refined structure.

**Fig. 3.**
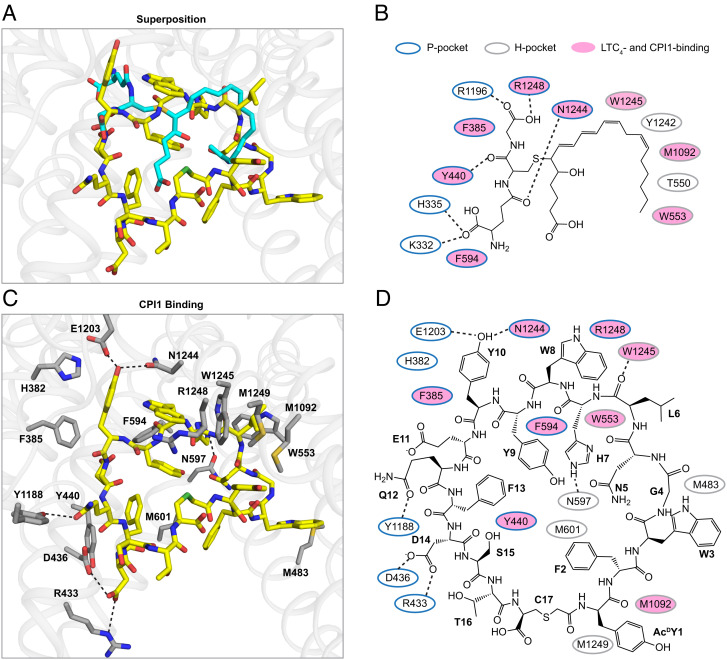
CPI1 binds at the same site as LTC_4_. (*A*) Local superposition of the CPI1- (yellow) and LTC_4_- (cyan) bound structures based on TM helices 6, 7, 8, 11, 15, and 16. MRP1 is shown as grey ribbons. (*B*) Diagram of MRP1 interactions with LTC4. Dashed lines represent hydrogen bonds and salt bridges. Residues forming the P-pocket are circled in blue and the H-pocket residues are in grey. Residues that also interact with CPI1 are indicated in pink. (*C*) Zoomed-in view of the CPI1-binding site with interacting side chains shown as sticks. Hydrogen bonds and salt bridges are indicated by dashed lines. Residues that form van der Waals contacts with CPI1 are also indicated. (*D*) Diagram of the CPI1 interactions with MRP1, annotated as in panel *B*.

The overall structure of peptide-inhibited MRP1 exhibits an NBD-separated, inward-facing conformation (Fig. 2 *B* and *C*). CPI1 binds at the interface of the two transmembrane helical bundles, filling almost the entire transmembrane pathway. The cyclic peptide exhibits a puckered annular conformation, stabilized by two intramolecular hydrogen bonds (Fig. 2*D*). Residues 1 to 13 of CPI are buried at its interface with MRP1, where they engage in extensive hydrogen bonds and van der Waals interactions (Figs. 2*E* and 3). Residues 15 to 17 make no contact with the transporter and are more flexible, as evidenced by the weaker density in this region (*SI Appendix*, Fig. S2). The two terminal residues, Ac^D^Y at position 1 (Ac^D^Y1) and cysteine at position 17 (C17), are connected via a thioether bond. This cyclic link is exposed to solvent (Fig. 2*E*), consistent with how this region was tethered to a 3′ puromycin moiety via C17 for affinity screening in the RaPID protocol (Fig. 1*A*).

The superposition of the CPI1-bound MRP1 structure with the LTC_4_-bound structure shows that CPI1 binds to the same site as LTC_4_ and interacts with many of the same residues (Fig. 3). The substrate-binding site of MRP1 has been described as bipartite, comprising a positively charged P-pocket that coordinates the glutathione (GSH) moiety on LTC_4_ and a largely hydrophobic H-pocket that surrounds the lipid tail (Fig. 3*B*). CPI1 interacts with residues in both the P- and H-pockets and, because of its large size, engages additional residues toward the cytoplasmic opening of the transmembrane pathway (Fig. 3*C*). The molecular contacts between CPI1 and MRP1 include seven aromatic–aromatic interactions, six methionine–aromatic interactions, seven hydrogen bonds, and a cation–aromatic interaction (Fig. 3 *C* and *D*). Of the 17 residues interacting with CPI1, eight are also involved in LTC_4_ recognition (Fig. 3 *B* and *D*). The solvent-accessible surface area of MRP1 that is buried by CPI1 is 1,580 Å^2^, about two times that of the binding surface of LTC_4_ (796 Å^2^). The larger interface and additional molecular interactions explain the higher affinity of CPI1 (~100 nM) compared to LTC_4_ (~350 nM) (20) (Fig. 1*D*) and thus render CPI1 a potent competitive inhibitor of LTC_4_. All residues involved in CPI1 binding are conserved between bMRP1 and hMRP1, suggesting that CPI1 is likely to interact with hMRP1 in a very similar manner.

### CPI1 Traps MRP1 in an Apo-Like Conformation.

**Fig. 4.**
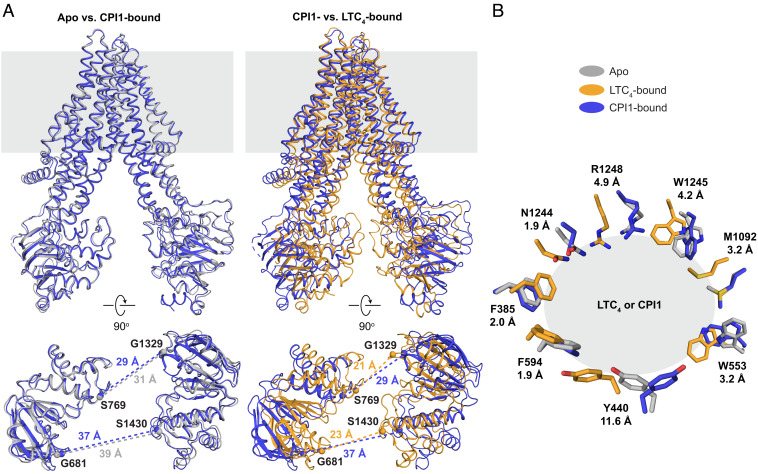
Binding of CPI1 and LTC_4_ stabilize MRP1 in different conformations. (*A*) Superposition of CPI1-bound structure (blue) with the apo (grey, *Left*) and LTC_4_-bound (orange, *Right*) structures. (*Top*) The overall structures. (*Bottom*) The NBD dimer. The distances between the Cα atoms of the Walker A glycine and signature motif serine on opposing NBDs are indicated. (*B*) Eight residues interact with both CPI1 and LTC_4_. Distances indicate the largest displacement of each side chain between LTC_4_-bound and CPI1-bound conformations.

To gain deeper insight into the mechanism by which CPI1 inhibits MRP1, we compared the structure of the CPI1-bound complex with those of the apo and LTC_4_-bound conformations. The overall root mean square deviation (rmsd) between the apo and CPI1-bound structures (approximately 1 Å) is much smaller than between the CPI1- and LTC_4_-bound structures (approximately 4.5 Å) (Fig. 4*A*). CPI1 stabilizes MRP1 in an apo-like conformation, in which the two NBDs are well separated and twisted relative to each other (Fig. 4 *A*, *Left*). The two halves of the transporter are closer in the LTC_4_-bound structure and the NBDs separated more evenly (Fig. 4 *A*, *Right*). These conformational differences are consistent with CPI1 inhibition and LTC_4_ stimulation of the ATPase activity of MRP1 (Fig. 1).

The structural plasticity of the substrate-binding site is evident by the local conformational changes that result from ligand binding (Fig. 4*B*). For example, Y440 undergoes a 5 Å translation between the apo and LTC_4_-bound structures to form a hydrogen bond with the γ-glutamate of the GSH moiety. In the CPI1-bound structure, however, the tyrosine hydroxyl of Y440 is displaced ~12 Å from its position in the LTC_4_-bound conformation and forms an aromatic–aromatic interaction with F13. Other examples include W553 and W1245, whose side chains form a parallel “tryptophan sandwich” with the lipid tail of LTC_4_ but adopt very different orientations when interacting with CPI1 (Fig. 4*B*). The functional importance of these residues has been established in mutational studies, in which mutation of Y440, W553, or W1245 severely reduced or eliminated MRP1-mediated transport of multiple cytotoxic organic anions (43–45).

## Discussion

MRP1 affects the distribution of a diverse range of endobiotics and xenobiotics throughout the human body. Selective modulation of MRP1 may therefore be a useful therapeutic strategy to treat multiple human diseases. In this study, we identified a cyclic peptide, CPI1, that inhibits MRP1 with nanomolar potency but shows minimal inhibition of Pgp. CPI1 competes with LTC_4_ for the same binding site, despite their very different molecular structures. Whereas CPI1 is a cyclic peptide of 17 amino acids, LTC_4_ is a cysteinyl leukotriene consisting of an arachidonic acid derivative conjugated to the tripeptide GSH.

All eight MRP1 residues that interact with both CPI1 and LTC_4_ have large side chains that can participate in a wide variety of van der Waals and electrostatic interactions. For example, the side chains of N1244 and R1248 contain multiple hydrogen bond donor and acceptor groups. The three amphipathic aromatic residues, Y440, W553, and W1245, can form nonpolar, hydrogen bond, and cation–π interactions. The two phenylalanine residues, F385 and F594, can participate in a variety of interactions due to their hydrophobic π–electron aromatic surface. Finally, M1092 contains a highly flexible side chain known to promote promiscuous binding (46–48). Thus, the substrate-binding site of MRP1 is enriched with residues that can form a variety of interactions with ligands. In addition, the global structure of MRP1 is intrinsically flexible, sampling multiple inward-facing states that may accommodate substrates of different sizes (22). The combination of this unique physicochemistry and structural plasticity of the substrate-binding site renders MRP1 a versatile host for a wide range of ligand molecules.

**Fig. 5.**
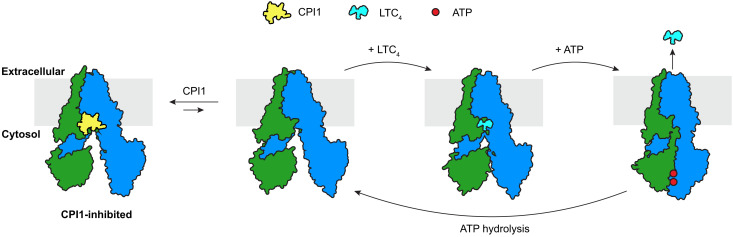
CPI1 arrests MRP1 in the inward-facing conformation while competing with LTC_4_. CPI1 is a competitive inhibitor of LTC_4_ that stabilizes MRP1 in the inward-facing conformation (left diagram), preventing ATP-dependent LTC_4_ export (transport cycle depicted on the right). Colors as in Figs. 2 and 3.

Although LTC_4_ and CPI1 bind to the same site on the transporter, LTC_4_ is a substrate whereas CPI1 is an inhibitor. The molecular mechanism by which LTC_4_ binding and ATP hydrolysis yield an efficient transport cycle is well understood (Fig. 5) (20–22). LTC_4_ induces a large conformational change that brings the NBDs near one another, priming them for dimerization. ATP binding induces further conformational changes that bring the two NBDs into contact with each other, poising them for ATP hydrolysis. These conformational changes open the translocation pathway to the extracellular space and reshape the structure of the substrate-binding site such that the affinity for LTC_4_ is reduced. As a result, substrate is ejected, and ATP is hydrolyzed. In contrast, CPI1 stabilizes MRP1 in an apo-like conformation in which the two NBDs are well separated and misaligned (Fig. 5). Because of its large size, CPI1 sterically obstructs the large conformational changes that are required for reorientation of the translocation pathway to an outward-facing conformation. In this manner, CPI1 traps the transporter in an inward-facing, ATPase incompetent state.

This inhibitory mechanism is reminiscent of that utilized by the herpes simplex virus inhibitory protein ICP47 on the transporter associated with antigen processing (TAP) (49). Like MRP1, TAP is an ABC transporter that alternates between inward- and outward-facing conformations. It is expressed in the endoplasmic reticulum, where it is responsible for delivering peptide antigens from the cytosol to the ER lumen for presentation to the immune system. ICP47 is a small protein deployed by the herpes simplex virus to evade immune surveillance. It binds to and inhibits TAP to avoid presentation of viral peptides to the immune system, allowing the virus to go undetected. Similar to CPI1, ICP47 inserts into the translocation pathway of TAP, blocking substrate binding and trapping TAP in an inward-facing conformation (49).

Thus, the strategy evolved by a persistent virus can be recapitulated by a synthetic peptide to regulate the activity of a different ABC transporter. In the case of CPI1, inhibition of MRP1 may ultimately benefit humans by facilitating drug delivery across biological barriers, thus improving the efficacy of chemotherapy. To further investigate the therapeutic potential of CPI1, several issues remain to be addressed. As CPI1 binds at the intracellular side of MRP1, it must cross the cellular membrane to achieve a therapeutic effect. With eight out of 17 CPI1 residues being polar, CPI1 in its present form is unlikely to be membrane permeable. Strategies such as saturating mutagenesis and N-methylation (50–52) can be explored to enhance passive membrane permeability. Alternatively, cell-penetrating peptides can be fused to CPI1 to promote active cellular uptake (53). Another consideration is specificity. Although CPI1 is selective against Pgp, it may inhibit other closely related transporters such as MRP2 and MRP3, whose substrate spectrum overlaps with that of MRP1 (7) and many of the CPI1 interacting residues are conserved. Counterscreening against these transporters will facilitate selecting subtype-specific inhibitors.

## Materials and Methods

### Cell Culture.

*Spodoptera frugiperda* Sf9 cells in suspension were passaged in Sf-900 II serum-free media (Gibco) with 5% fetal bovine serum (FBS) at 27 °C in an orbital shaker. HEK293S GnTI- cells in suspension were passaged in Freestyle 293 media (Gibco) supplemented with 2% FBS at 37 °C, 8% CO_2_, and 80% humidity in an orbital shaker.

### Protein Expression and Purification.

Bovine and human MRP1, as well as human Pgp, were expressed and purified as described previously. Briefly, mammalian codon-optimized genes (BioBasic Inc.) were cloned into a plasmid vector in-frame with a PreScission Protease cleavage site and a C-terminal eGFP tag. Plasmid vectors were transformed into DH10Bac cells for production of recombinant bacmid, which was subsequently purified and transfected into Sf9 cells using Cellfectin II reagent (ThermoFisher) to make baculovirus. After serial amplification in Sf9 cells, baculovirus was added at 10% v/v to HEK293S GnTl^-^ cells in suspension at a density of 2.5 to 3 million cells/mL at 37 °C. 10 mM sodium butyrate was added after 12 h, and the temperature dropped to 30 °C. Cells were pelleted 48 h later, washed once with phosphate buffered saline (PBS), flash-frozen in liquid nitrogen, and maintained at −80 °C until protein purification.

All protein purification steps were performed on ice or at 4 °C. Thawed cell pellets were resuspended with a handheld homogenizer (Polytron PT1200E) in a solution of 300 mM NaCl, 50 mM HEPES pH 8.0, 2 mM MgCl_2_, 2 mM dithiothreitol (DTT), and 20% glycerol with DNase I and protease inhibitors (1 µg/mL aprotinin, 0.6 mM benzamidine, 1 µg/mL leupeptin, 1 µg/mL pepstatin A, 1 mM phenylmethylsulfonyl fluoride, and 100 µg/mL soy trypsin inhibitor). Membranes were solubilized for 1.5 h by addition of 2% lauryl maltose neopentyl glycol and 0.2% cholesteryl hemisuccinate, after which insoluble material was removed by centrifugation for 40 min at 75,000 × g. Supernatant was mixed with GFP nanobody-conjugated Sepharose 4 Fast Flow resin (GE Healthcare) for 1.5 h, after which the slurry was packed into a column and washed with a solution of 0.06% digitonin, 150 mM NaCl, 50 mM HEPES pH 8.0, 2 mM MgCl_2_, 2 mM DTT, and 5% glycerol. PreScission protease was added to the column at a concentration of 0.35 mg/mL for 3 h to liberate MRP1 or Pgp from the GFP-nanobody resin. Eluate was passed through a glutathione sepharose resin to remove PreScission protease and concentrated with a 100 kDa molecular weight cutoff centrifugal filter. Concentrate was loaded onto a GE Healthcare Superose 6 size exclusion column equilibrated in 0.06% digitonin, 150 mM KCl, 50 mM Tris pH 8.0, 2 mM MgCl_2_, and 2 mM DTT. Peak fractions were collected, pooled, and concentrated for ATPase assays or freezing cryo-EM grids.

### Screening of hMRP1 Binding Macrocyclic Peptides with the RaPID System.

In vitro RaPID selection of hMRP1 binding macrocyclic peptides was performed as previously reported with slight modifications. Briefly, the initial random DNA library was transcribed and ligated to a puromycin linker primer via T4 ligase for 30 min at 25 °C after which it was extracted with phenol/chloroform and precipitated with ethanol. A 150 μL translation reaction using the methionine-deficient FIT system and a 50 μM concentration of aminoacylated initiator tRNA. ClAc-L-Tyr-tRNA^fMet^_CAU_ and ClAc-D-Tyr-tRNA^fMet^_CAU_ were used to convert the mRNA library into a library of peptide-mRNA fusions. In vitro translation was performed at 37 °C for 30 min followed by a 12-min incubation at 25 °C to enhance the formation of the peptide–mRNA fusions. Thirty microliters of 100 mM EDTA was added to dissociate ribosomes to release peptides, and the mixture was incubated at 37 °C for 30 min to allow the thioether cyclization to complete. The peptide-fused mRNA was subsequently reverse-transcribed using MMLV RT RNase H (Promega) for 1 h at 42 °C to form peptide-cDNA fusion and 0.5 μL aliquot of this mixture was taken and saved for the determination of the total amount of inputted cDNA (mRNA). The peptide–cDNA fusions were then incubated with human MRP1 protein immobilized on anti-GFP nanobody-coated Sepharose beads via GFP tag for 30 min at 4 °C with mixing. Beads were washed with cold protein buffer by centrifugation and removing supernatant three times after which bound cDNAs were eluted by mixing the beads with 1× PCR buffer and heating at 95 °C for 5 min, followed by immediate separation of the supernatant from the beads. A small fraction of the cDNA from the mixture was taken and used in real-time PCR for quantification using a LightCycler 2.0 (Roche). The remainder of the mixture was amplified by PCR. The resulting DNAs were purified by phenol–chloroform extraction and ethanol precipitation and transcribed into mRNAs for the next round of selection. From the second round of selection, the translation was performed at 5 μL scale, and three negative selections were performed before the positive selection step using only anti-GFP nanobody-coated Sepharose beads. Finally, the observed enrichments appearing at seventh round were subjected to further DNA deep sequencing using the MiSeq sequencing system (Illumina).

### Chemical Synthesis of Peptides.

Macrocyclic peptides were synthesized using standard Fmoc solid-phase peptide synthesis using a Syro Wave automated peptide synthesizer (Biotage). After completion of synthesis, resin (25 μmol scale) was treated with a solution of 92.5% trifluoroacetic acid (TFA), 2.5% water, 2.5% triisopropylsilane, and 2.5% ethanedithiol, to affect global deprotection and cleavage of the free linear N-ClAc-peptide. Peptide was precipitated from the cleavage mixture by adding cold diethyl ether. After three rounds of washing with cold ether, the pellet was dissolved in 5 mL DMSO, basified with Diisopropylethylamine, and incubated for 2 h at 25 °C, to yield the corresponding macrocycle. The peptide solutions were then acidified with TFA to quench the macrocyclization reaction. The resulting macrocycles were purified by reverse phase preparative High Performance Liquid Chromatography (HPLC), using a Prominence HPLC system (Shimadzu) under linear gradient conditions. Mobile-phase A (comprising water with 0.1% TFA) was mixed with mobile-phase B (0.1% TFA in acetonitrile). Purified peptides were lyophilized in vacuo and molecular mass was confirmed by MALDI MS, using an AutoFlex II instrument (Bruker Daltonics).

### ATPase Assay.

ATPase activity was measured by recording the rate of NADH consumption during ATP regeneration from ADP. MRP1 or Pgp was added at concentration of 0.5 µM or 1 µM where indicated to a reaction mixture containing 60 µg/mL pyruvate kinase, 32 µg/mL lactate dehydrogenase, 9 mM phosphoenolpyruvate, and 150 µM nicotinamide adenine dinucleotide (NADH) in size exclusion buffer. MRP1 substrates and cyclic peptides in 100% dimethyl sulfoxide (DMSO) were added such that the final DMSO concentration did not exceed 2.5%, and 4 mM ATP-Mg^2+^ was added to initiate the reaction. NADH consumption was measured by monitoring the rate of NADH fluorescence depletion (λ_ex_/λ_em_ 340/445 nm) using an Infinite M1000 microplate reader (Tecan) at 28 °C. ATPase activity was quantified by calculating the mean and SEM of at least three separate measurements from a single protein purification for each of bMRP1, hMRP1, and hPgp. Data were fit by nonlinear regression to the Michaelis–Menten equation using GraphPad Prism (Dotmatics). The inhibition constant $K_{i}$ for CPI1 was determined though fitting the reaction data to a modified version of the quadratic binding equation to account for ligand depletion at relatively high (1 µM) MRP1 concentrations:

$$\begin{array}{c} K_{i}\;=\;\frac{[R]\left[L\right]}{\left[R\cdot L\right]}=\;\frac{\left([R{]}_{\mathrm{tot}}-[R\cdot L]\right)\left([L{]}_{\mathrm{tot}}-[R\cdot L]\right)}{\left[R\cdot L\right]}, \end{array}$$

$$\begin{array}{c} K_{i}\;=\;\frac{[R{]}_{\mathrm{tot}}\;\times \;[L{]}_{\mathrm{tot}}\;-\;\left([L{]}_{tot}\;+\;[R{]}_{tot}\right)[R\cdot L]\;+\;[R\cdot L{]}^{2}}{\left[R\cdot L\right]}, \end{array}$$

$$[R\cdot L]K_{i}\;=\;[R{]}_{tot}\;\times \;[L{]}_{tot}\;-\;([L{]}_{tot}\;+\;[R{]}_{tot})[R\cdot L]\;+\;[R\cdot L{]}^{2},\;$$

$$\begin{array}{c} 0\;=\;[R{]}_{tot}\;\times \;[L{]}_{\mathrm{tot}}\;-\;([L{]}_{tot}\;+\;[R{]}_{tot}\;+\;K_{i})[R\cdot L]\;+\;[R\cdot L{]}^{2}, \end{array}$$

$$\begin{array}{c} {}[R\cdot L]\;=\;\frac{([L{]}_{tot}\;+\;[R{]}_{tot}\;+\;K_{i})\;-\;\sqrt{([L{]}_{tot}\;+\;[R{]}_{tot}\;+\;K_{i}{)}^{2}\;-\;4\;\times \;\left([R{]}_{tot}\;\times \;[L{]}_{tot}\right)}}{2} \end{array},$$

$$Y=\frac{\left[R\cdot L\right]}{[R{]}_{tot}}\;,\;$$

$$\begin{array}{c} Y\;=\;\frac{([L{]}_{tot}\;+\;[R{]}_{tot}\;+\;K_{i})\;-\;\sqrt{\left([L{]}_{tot}\;+\;[R{]}_{tot}\;+\;K_{i}{)}^{2}\;-\;4\;\times \;([R{]}_{tot}\;\times \;[L{]}_{tot}\right)}}{2\;\times \;[R{]}_{\mathrm{tot}}}\;, \end{array}$$

$$Range\;normalization\;factor\;=\;(V_{max}\;-\;V_{min})\;$$

$$\begin{array}{c} V\;=\;V_{\max}\;-\;(V_{max}\;-\;V_{min})\;\times \;\\ \;\frac{([L{]}_{tot}\;+\;[R{]}_{tot}\;+\;K_{i})\;-\;\sqrt{\left([L{]}_{tot}\;+\;[R{]}_{tot}\;+\;K_{i}{)}^{2}\;-\;4\;\times \;([R{]}_{tot}\;\times \;[L{]}_{tot}\right)}}{2\;\times \;[R{]}_{tot}}\;, \end{array}$$

where $[L{]}_{tot}$ is the concentration of CPI1, $[R{]}_{tot}$ is the concentration of MRP1, $V_{\max}$ is the maximal ATPase activity in the absence of CPI1, and $V_{\min}$ is the ATPase activity in the presence of saturating CPI1.

### Cryo-EM Sample Preparation and Data Collection.

Freshly purified bMRP1 at a concentration of 5 µM (0.86 mg/mL) was incubated with 12.5 µM cyclic peptide on ice for 30 min with a final DMSO concentration of 2.5%. The bMRP1-cyclic peptide sample was then concentrated using a 100 kDa MWCO centrifugal filter to 4.5 mg/mL, and 3 mM fluorinated Fos-Choline-8 was added immediately prior to cryo-EM grid freezing. The sample solution was applied to freshly glow-discharged Quantifoil R1.2/1.3 400-mesh Au Holey Carbon Grids before freezing in liquid ethane using a Vitrobot Mark IV (FEI). Final bMRP1 protein concentrations were approximately 4 mg/mL.

Cryo-EM data were obtained using a Titan Krios electron microscope (FEI) operated at 300 kV with a K2 Summit camera (Gatan) operated in super-resolution mode (0.515 Å/pixel). Data acquisition was automated through SerialEM. The electron dose rate was 8 e^−^/pixel/sec over an exposure time of 10 s, divided into 50 subframes. A total of 3,708 movies were collected in a single dataset from one grid (*SI Appendix*, Table S1).

### Cryo-EM Data Processing.

Individual movie frames were imported into RELION for processing (*SI Appendix*, Fig. S1). Images were corrected with a gain reference and binned by two for a pixel size of 1.03 Å/pixel. Subframe image alignment was performed though MotionCor2. The contrast transfer function (CTF) was estimated with CTFFIND4 and each micrograph was inspected individually. Two-dimensional (2D) classes from a previous bMRP1-apo dataset were used as templates to autopick particles in RELION from a subset of micrographs. Autopicked particles underwent 2D classification, and the best 2D classes from the bMRP1-CPI1 initial model were used as templates to autopick particles from all micrographs in the dataset, yielding a total of 805,615 particles (*SI Appendix*, Fig. S1). These particles were subjected to 2D classification, after which the 547,189 particles in the best classes underwent three-dimensional (3D) classification into four classes in RELION. A semicircular region of density was clearly visible in the substrate binding site of the dominant 3D class (34.9%, 8.0 Å). The 214,651 particles in this class were run-through a two-stage 3D refinement in RELION. Initial 3D refinement was carried out with a loose mask including the micelle and all domains of bMRP1. Subsequent 3D refinement was continued with a smaller mask excluding the micelle and density corresponding to TMD0. Iterations of postprocessing, CTF refinement, Bayesian polishing, and masked 3D refinement were performed, followed by a 3D classification without alignment incorporating the smaller mask excluding the micelle and TMD0. This yielded a best class of 134,960 particles. These particles were subjected to additional iterations of postprocessing, CTF refinement, Bayesian polishing, and masked 3D refinement. The resolution of the final 3.27 Å map was determined from the Fourier shell correlation (FSC) of two reconstructions each generated from half of the data and a cutoff criterion of 0.143. Estimations of local resolution were performed using Blocres. Other classes, despite having lower resolution, have very similar overall structure and similar degree of NBD separation as the 3.27 Å reconstruction.

### Model Building, Refinement, and Model Validation.

The atomic coordinates for apo bMRP1 (5UJ9) were used as a starting point for model building. The coordinates were positioned into a P1 crystallographic unit cell with 5 Å padding around the model using pdbset, and the bMRP1 + CPI1 density map was positioned into an identical unit cell using Maprot. A noncrystallographic symmetry mask was generated using ncsmask, and Sfall was used to calculate structure factors and phases from the repositioned map. These operations were applied to the full density map and two half maps (working and free), the former of which was used for model building and refinement. The structure was manually fit to the bMRP1 + CPI1 working map in Coot, and real-space refined against the working map in the PHENIX suite. Building of CPI1 into the EM density in the MRP1 substrate binding site was facilitated through generation of a density-modified map in the PHENIX suite using a polyalanine model of bMRP1 lacking any ligands (54). A fully cyclic region of density is clearly visible in the MRP1 in this density-modified map contoured to 1.00 RMSD (*SI Appendix*, Fig. S2).

To generate an atomic model of CPI1, the SMILES string corresponding to the chemical structure of CPI1 was first used to generate an initial model and restraints in AceDRG. This initial model was positioned into the cyclic density in the MRP1 substrate binding site with restraints in Coot and merged with the coordinate file containing the bMRP1 atomic model lacking TMD0 refined against the original half-map. The merged coordinates were iteratively refined against the density-modified map using REFMAC incorporating secondary structure restraints generated with ProSMART, and manually manipulated to fit the density-modified map in Coot. The final model demonstrates excellent structural characteristics as analyzed by MolProbity (*SI Appendix*, Fig. S2 and Table S1).

The atomic model was converted to a density map (model map) using UCSF Chimera, and Spider was used to determine the Fourier shell correlation (FSC) between the model map, the unprocessed full map, and the two unprocessed half-maps (working and free). All unprocessed maps were given a mask with a smooth edge about 3.5 times that of the model-map, and the FSC curves for the unprocessed maps were then adjusted for the volume exceeding that of the model map by the equation

$${\mathrm{FSC}}_{\mathrm{corrected}}\;=\;\frac{f\;\times \;FSC}{1\;+\;(f\;-\;1)\;\times \;FSC},$$

in which f is the factor by which the masked map volume exceeds that of the model map. The FSC for the model map versus the full map equals 0.5 at a resolution of 3.20 Å (*SI Appendix*, Fig. S2). Rwork and Rfree values were calculated using REFMAC with a mask containing the model plus a 2 Å margin against the two half maps. Model and map figure panels were generated using PyMOL.

## Supplementary Material

Appendix 01 (PDF)

## Data Availability

Atomic coordinates have been deposited in the Protein Data Bank (accession number 8F4B). Cryo-EM density maps have been deposited in the Electron Microscopy Data Bank (accession number EMD-28854).

## References

[1] M. J. Flens , Tissue distribution of the multidrug resistance protein. Am. J. Pathol. **148**, 1237–1247 (1996). 8644864 PMC1861521

[2] A. C. Jaramillo, F. A. Saig, J. Cloos, G. Jansen, G. J. Peters, How to overcome ATP-binding cassette drug efflux transporter-mediated drug resistance? Cancer Drug Resist. **1**, 6–29 (2018).

[3] M. Kourti , Expression of multidrug resistance 1 (MDR1), multidrug resistance-related protein 1 (MRP1), lung resistance protein (LRP), and breast cancer resistance protein (BCRP) genes and clinical outcome in childhood acute lymphoblastic leukemia. Int. J. Hematol. **86**, 166–173 (2007). 17875533 10.1532/IJH97.E0624

[4] J. M. Bréchot, I. Hurbain, A. Fajac, N. Daty, J. F. Bernaudin, Different pattern of MRP localization in ciliated and basal cells from human bronchial epithelium. J. Histochem. Cytochem. **46**, 513–517 (1998). 9524197 10.1177/002215549804600411

[5] J. F. Lu, D. Pokharel, M. Bebawy, MRP1 and its role in anticancer drug resistance. Drug Metab. Rev. **47**, 406–419 (2015). 26541366 10.3109/03602532.2015.1105253

[6] R. G. Deeley, S. P. C. Cole, Substrate recognition and transport by multidrug resistance protein 1 (ABCC1). FEBS Lett. **580**, 1103–1111 (2006). 16387301 10.1016/j.febslet.2005.12.036

[7] R. G. Deeley, C. Westlake, S. P. C. Cole, Transmembrane transport of endo- and xenobiotics by mammalian ATP-binding cassette multidrug resistance proteins. Physiol. Rev. **86**, 849–899 (2006). 16816140 10.1152/physrev.00035.2005

[8] E. M. Leslie, R. G. Deeley, S. P. C. Cole, Multidrug resistance proteins: Role of P-glycoprotein, MRP1, MRP2, and BCRP (ABCG2) in tissue defense. Toxicol. Appl. Pharmacol. **204**, 216–237 (2005). 15845415 10.1016/j.taap.2004.10.012

[9] M. Eilers, U. Roy, D. Mondal, MRP (ABCC) transporters-mediated efflux of anti-HIV drugs, saquinavir and zidovudine, from human endothelial cells. Exp. Biol. Med. (Maywood) **233**, 1149–1160 (2008). 18535159 10.3181/0802-RM-59 PMC2575034

[10] B. G. Peterson, K. W. Tan, B. Osa-Andrews, S. H. Iram, High-content screening of clinically tested anticancer drugs identifies novel inhibitors of human MRP1 (ABCC1). Pharmacol. Res. **119**, 313–326 (2017). 28258008 10.1016/j.phrs.2017.02.024

[11] X. Zhao , Involvement of human and canine MRP1 and MRP4 in benzylpenicillin transport. PLoS One **14**, e0225702 (2019). 31774876 10.1371/journal.pone.0225702 PMC6881026

[12] J. Li , Expression of MRP1, BCRP, LRP, and ERCC1 in advanced non–small-cell lung cancer: Correlation with response to chemotherapy and survival. Clin. Lung Cancer **10**, 414–421 (2009). 19900859 10.3816/CLC.2009.n.078

[13] M. A. Barrand, T. Rhodes, M. S. Center, P. R. Twentyman, Chemosensitisation and drug accumulation effects of cyclosporin A, PSC-833 and verapamil in human MDR large cell lung cancer cells expressing a 190k membrane protein distinct from P-glycoprotein. Eur. J. Cancer **29**, 408–415 (1993). 10.1016/0959-8049(93)90397-x 8398342

[14] A. Larkin , Investigation of MRP-1 protein and MDR-1 P-glycoprotein expression in invasive breast cancer: A prognostic study. Int. J. Cancer **112**, 286–294 (2004). 15352042 10.1002/ijc.20369

[15] L. O'driscoll , MDR1/P-glycoprotein and MRP-1 drug efflux pumps in pancreatic carcinoma. Anticancer Res. **27**, 2115–2120 (2007). 17695494

[16] X. Tong, J. Zhao, Y. Zhang, P. Mu, X. Wang, Expression levels of MRP1, GST-π, and GSK3β in ovarian cancer and the relationship with drug resistance and prognosis of patients. Oncol. Lett. **18**, 22–28 (2019). 31289467 10.3892/ol.2019.10315 PMC6540457

[17] N. Walsh , Membrane transport proteins in human melanoma: Associations with tumour aggressiveness and metastasis. Br. J. Cancer **102**, 1157–1162 (2010). 20234362 10.1038/sj.bjc.6605590 PMC2853088

[18] M. D. Norris , Expression of the gene for multidrug-resistance–associated protein and outcome in patients with neuroblastoma. N. Engl. J. Med. **334**, 231–238 (1996). 8532000 10.1056/NEJM199601253340405

[19] K. K. Matthay , Neuroblastoma. Nat. Rev. Dis. Primers **2**, 1–21 (2016). 10.1038/nrdp.2016.78 27830764

[20] Z. L. Johnson, J. Chen, Structural basis of substrate recognition by the multidrug resistance protein MRP1. Cell **168**, 1075–1085.e9 (2017). 28238471 10.1016/j.cell.2017.01.041

[21] Z. L. Johnson, J. Chen, ATP binding enables substrate release from multidrug resistance protein 1. Cell **172**, 81–89.e10 (2018). 29290467 10.1016/j.cell.2017.12.005

[22] L. Wang , Characterization of the kinetic cycle of an ABC transporter by single-molecule and cryo-EM analyses. eLife **9**, 1–20 (2020). 10.7554/eLife.56451 PMC7253176 32458799

[23] J. Wijnholds , Increased sensitivity to anticancer drugs and decreased inflammatory response in mice lacking the multidrug resistance-associated protein. Nat. Med. **3**, 1275–1279 (1997). 9359705 10.1038/nm1197-1275

[24] I. Leier, G. Jedlitschky, U. Buchholz, D. Keppler, Characterization of the ATP-dependent leukotriene C4 export carrier in mastocytoma cells. Eur. J. Biochem. **220**, 599–606 (1994). 8125120 10.1111/j.1432-1033.1994.tb18661.x

[25] M. Qadir , Cyclosporin A is a broad-spectrum multidrug resistance modulator. Clin. Cancer Res. **11**, 2320–2326 (2005). 15788683 10.1158/1078-0432.CCR-04-1725

[26] A. H. Dantzig , Evaluation of the binding of the tricyclic isoxazole photoaffinity label LY475776 to multidrug resistance associated protein 1 (MRP1) orthologs and several ATP-binding cassette (ABC) drug transporters. Biochem. Pharmacol. **67**, 1111–1121 (2004). 15006547 10.1016/j.bcp.2003.11.006

[27] C. A. Burkhart , Small-molecule multidrug resistance-associated protein 1 inhibitor reversan increases the therapeutic index of chemotherapyin mouse models of neuroblastoma. Cancer Res. **69**, 6573–6580 (2009). 19654298 10.1158/0008-5472.CAN-09-1075 PMC2746061

[28] J. I. Lai, Y. J. Tseng, M. H. Chen, C. Y. F. Huang, P. M. H. Chang, Clinical perspective of FDA approved drugs with P-Glycoprotein inhibition activities for potential cancer therapeutics. Front. Oncol. **10** (2020). 10.3389/fonc.2020.561936 PMC7704056 33312947

[29] A. K. Malde, T. A. Hill, A. Iyer, D. P. Fairlie, Crystal structures of protein-bound cyclic peptides. Chem. Rev. **119**, 9861–9914 (2019). 31046237 10.1021/acs.chemrev.8b00807

[30] A. Zorzi, K. Deyle, C. Heinis, Cyclic peptide therapeutics: Past, present and future. Curr. Opin. Chem. Biol. **38**, 24–29 (2017). 28249193 10.1016/j.cbpa.2017.02.006

[31] S. S. Usmani, THPdb: Database of FDA-approved peptide and protein therapeutics. PLoS One **12**, e0181748 (2017) 28759605 10.1371/journal.pone.0181748 PMC5536290

[32] Y. Goto, T. Katoh, H. Suga, Flexizymes for genetic code reprogramming. Nat. Protoc. **6**, 779–790 (2011). 21637198 10.1038/nprot.2011.331

[33] T. Passioura , De novo macrocyclic peptide inhibitors of hepatitis B virus cellular entry. Cell Chem. Biol. **25**, 906–915.e5 (2018). 29779957 10.1016/j.chembiol.2018.04.011

[34] C. Nitsche , De novo discovery of nonstandard macrocyclic peptides as noncompetitive inhibitors of the zika virus NS2B-NS3 protease. ACS Med. Chem. Lett. **10**, 168–174 (2019). 30783498 10.1021/acsmedchemlett.8b00535 PMC6378662

[35] M. Nawatha , De novo macrocyclic peptides that specifically modulate Lys48-linked ubiquitin chains. Nat. Chem. **11**, 644–652 (2019). 31182821 10.1038/s41557-019-0278-x PMC7341950

[36] M. E. Otero-Ramirez , Macrocyclic peptides that inhibit Wnt signalling via interaction with Wnt3a. RSC Chem. Biol. **1**, 26–34 (2020). 34458746 10.1039/d0cb00016g PMC8382136

[37] A. Kawamura , Highly selective inhibition of histone demethylases by de novo macrocyclic peptides. Nat. Commun. **8**, 1–10 (2017). 28382930 10.1038/ncomms14773 PMC5384220

[38] D. Hazama , Macrocyclic peptide-mediated blockade of the CD47-SIRPα interaction as a potential cancer immunotherapy. Cell Chem. Biol. **27**, 1181–1191.e7 (2020). 32640189 10.1016/j.chembiol.2020.06.008

[39] K. Sakai , Macrocyclic peptide-based inhibition and imaging of hepatocyte growth factor. Nat. Chem. Biol. **15**, 598–606 (2019). 31101918 10.1038/s41589-019-0285-7

[40] T. Katoh, T. Sengoku, K. Hirata, K. Ogata, H. Suga, Ribosomal synthesis and de novo discovery of bioactive foldamer peptides containing cyclic β-amino acids. Nat. Chem. **12**, 1081–1088 (2020). 32839601 10.1038/s41557-020-0525-1

[41] E. Stefan , De novo macrocyclic peptides dissect energy coupling of a heterodimeric ABC transporter by multimode allosteric inhibition. eLife **10**, 1–24 (2021). 10.7554/eLife.67732 PMC8116058 33929325

[42] Y. Taguchi, K. Saeki, T. Komano, Functional analysis of MRP1 cloned from bovine. FEBS Lett. **521**, 211–213 (2002). 12067707 10.1016/s0014-5793(02)02816-8

[43] K. I. Ito, S. L. Olsen, W. Qiu, R. G. Deeley, S. P. C. Cole, Mutation of a single conserved tryptophan in multidrug resistance protein 1 (MRP1/ABCC1) results in loss of drug resistance and selective loss of organic anion transport. J. Biol. Chem. **276**, 15616–15624 (2001). 11278867 10.1074/jbc.M011246200

[44] C. E. Grant, M. Gao, M. K. DeGorter, S. P. C. Cole, R. G. Deeley, Structural determinants of substrate specificity differences between human multidrug resistance protein (MRP) 1 (ABCC1) and MRP3 (ABCC3). Drug Metab. Disposition **36**, 2571–2581 (2008). 10.1124/dmd.108.022491 18775981

[45] K. Koike , Multiple membrane-associated tryptophan residues contribute to the transport activity and substrate specificity of the human multidrug resistance protein, MRP1. J. Biol. Chem. **277**, 49495–49503 (2002). 12388549 10.1074/jbc.M206896200

[46] S. H. Gellman, On the role of methionine residues in the sequence-independent recognition of nonpolar protein surfaces. Biochemistry **30**, 6633–6636 (1991). 2065050 10.1021/bi00241a001

[47] H. D. Bernstein , Model for signal sequence recognition from amino-acid sequence of 54K subunit of signal recognition particle. Nature **340**, 482–486 (1989). 2502718 10.1038/340482a0

[48] K. T. O’Neil, W. F. DeGrado, How calmodulin binds its targets: Sequence independent recognition of amphipathic alpha helices. Trends Biochem. Sci. **15**, 59–64 (1990). 2186516 10.1016/0968-0004(90)90177-d

[49] M. L. Oldham , A mechanism of viral immune evasion revealed by cryo-EM analysis of the TAP transporter. Nature **529**, 537–540 (2016). 26789246 10.1038/nature16506 PMC4848044

[50] E. Biron , Improving oral bioavailability of peptides by multiple N-methylation: Somatostatin analogues. Angew. Chemie Int. Ed. **47**, 2595–2599 (2008). 10.1002/anie.200705797 18297660

[51] N. C. Tan, P. Yu, Y. U. Kwon, T. Kodadek, High-throughput evaluation of relative cell permeability between peptoids and peptides. Bioorganic Med. Chem. **16**, 5853–5861 (2008). 10.1016/j.bmc.2008.04.074 PMC2490712 18490170

[52] Y. U. Kwon, T. Kodadek, Quantitative comparison of the relative cell permeability of cyclic and linear peptides. Chem. Biol. **14**, 671–677 (2007). 17584614 10.1016/j.chembiol.2007.05.006

[53] S. H. Nam, J. Park, H. Koo, Recent advances in selective and targeted drug, gene delivery systems using cell-penetrating peptides. Arch Pharm. Res. **46**, 18–34 (2023). 36593377 10.1007/s12272-022-01425-y PMC9807432

[54] T. C. Terwilliger, S. J. Ludtke, R. J. Read, P. D. Adams, P. V. Afonine, Improvement of cryo-EM maps by density modification. Nat. Methods **17**, 923–927 (2020). 32807957 10.1038/s41592-020-0914-9 PMC7484085

