# Supplementary material for: A macrocyclic peptide inhibitor traps MRP1 in a catalytically incompetent conformation
Source: Proc Natl Acad Sci U S A. 2023 Mar 9;120(11):e2220012120. doi: 10.1073/pnas.2220012120 (PMC10089224; doi:10.1073/pnas.2220012120)
Supplement: Supplementary file 1 — Appendix 01 (PDF) [file pnas.2220012120.sapp.pdf]

Supplementary Materials for

A macrocyclic peptide inhibitor traps MRP1 in a catalytically incompetent conformation.

Harlan L. Pietz[1], Ata Abbas[2], Zachary Lee Johnson[1,3], Michael L. Oldham[1,4,5], Hiroaki Suga[2*], and Jue Chen[1,4,*]

[1]Laboratory of Membrane Biology and Biophysics, The Rockefeller University, New York, NY 10065, USA

[2]Department of Chemistry, School of Science, The University of Tokyo, Tokyo, Japan

[3]Present address: Schrödinger, Inc., New York, NY 10036, USA

[4]Howard Hughes Medical Institute, 1230 York Avenue, New York, NY 10065, USA

[5]Present address: Department of Structural Biology, St. Jude Children's Research Hospital, Memphis, TN 38105, USA

*Corresponding authors: Hiroaki Suga, Jue Chen.

Email: hsuga@chem.s.u-tokyo.ac.jp, juechen@rockefeller.edu

This PDF file includes:

Figures S1 and S2

Table S1

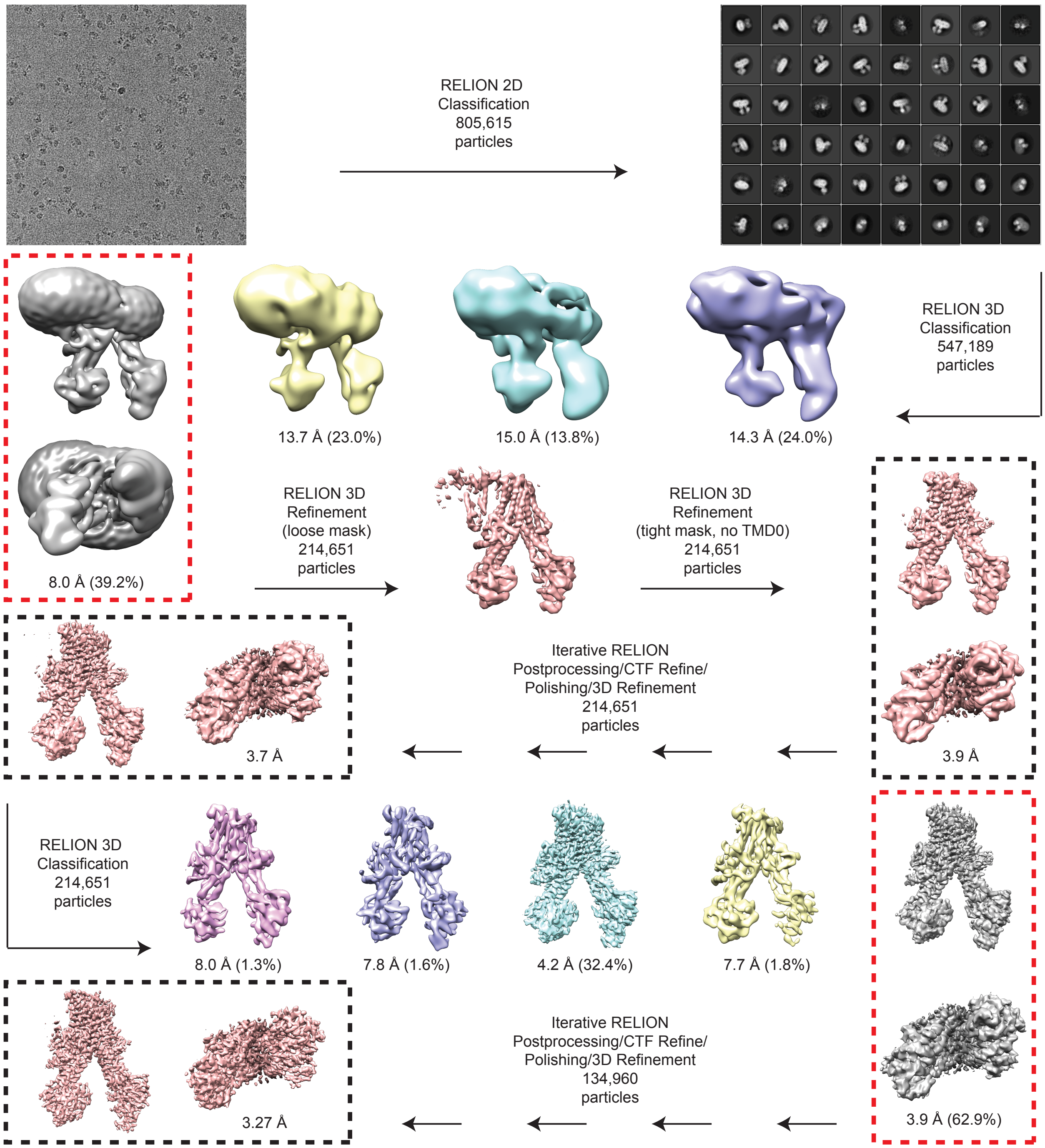

Figure S1. Cryo-EM data processing workflow. Image processing to yield the cryo-EM density map for initial MRP1 model building. A total of 134,960 particles were used for construction of the initial 3.27 Å map.

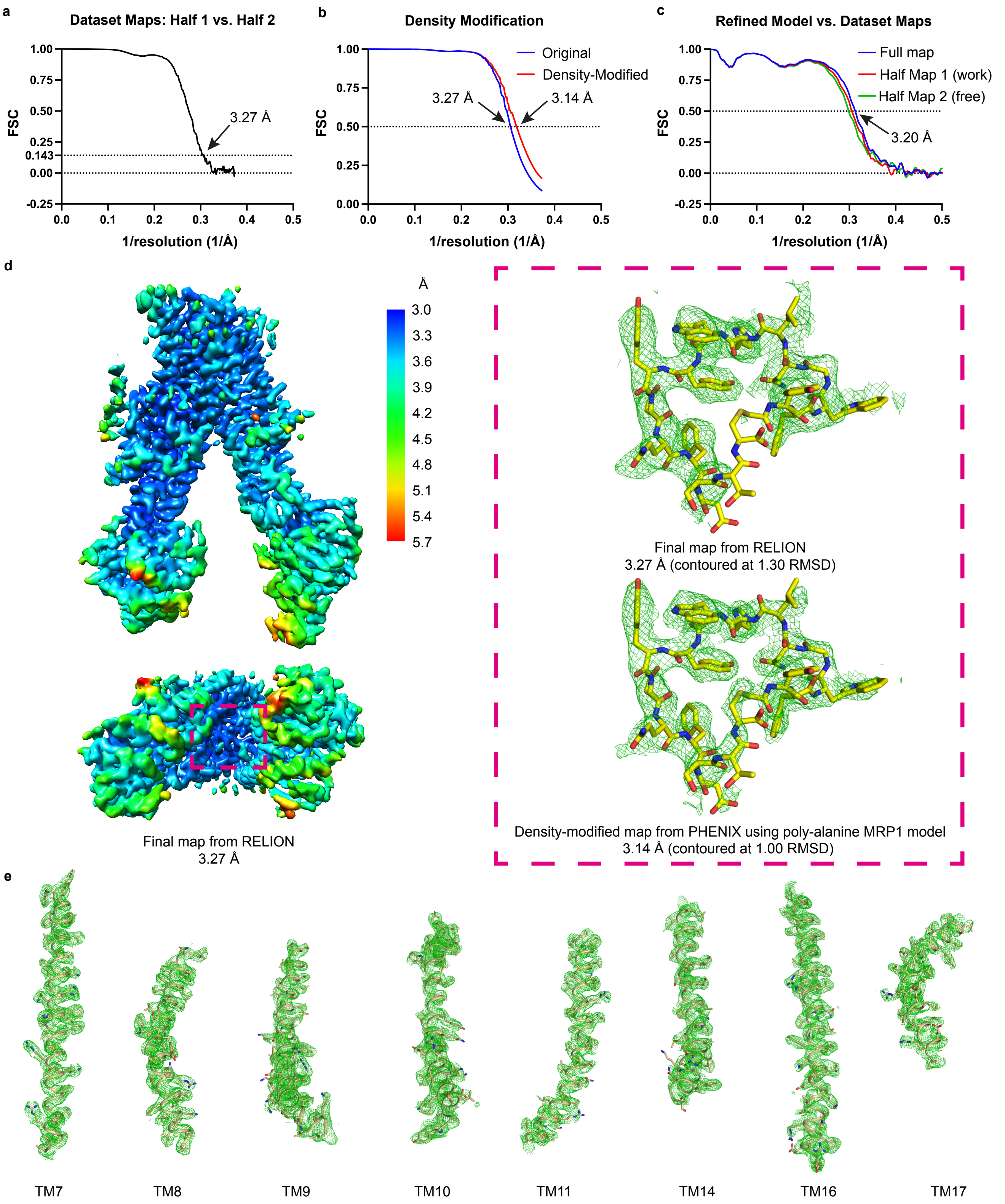

Figure S2. Cryo-EM map refinement and properties. a, Fourier shell correlation (FSC) between the two half maps leading to the initial 3.27 Å map. **b,** Comparison of the FSC between the initial 3.27 Å map and final density-modified map used for CPI1 model building and refinement. **c,** FSC comparison between the refined structure and the full map, the working half map, and free half map. **d,** Left: the initial 3.27 Å map colored by estimated local resolution (Blocres). Right: the initial 3.27 Å map (density, green mesh) was subjected to density modification (PHENIX) with a poly-alanine model of MRP1. **e,** Density for TM helices involved in CPI1 binding from the density-modified map.

**Data collection**	
Microscope	Titan Krios (FEI)
Voltage (kV)	300
Detector	K2 Summit (Gatan)
Pixel size (Å)	1.03
Defocus range (μM)	-0.7 to -2.4
Movies	3708
Frames/movie	50
Dose rate (electrons/pix/s)	8
Total dose (electrons/Å^2)	75.4
Number of particles	134960
**Model Composition**	
Chains	2
Non-hydrogen atoms	9488
Protein residues	1198
Lipids/detergents/ligands	1 (CPI1)
**Refinement (prior to density modification)**	
Resolution (Å)	3.27
Rwork	0.3397
Rfree	0.3496
RMS deviations	
Bond length (Å) (# > 4σ)	0.014 (0)
Bond angles (°) (# > 4σ)	1.697 (0)
**Validation**	
MolProbity score	1.18
Clash score	1.2
Ramachandran plot (%)	
Outliers	0
Allowed	4.97
Favored	95.03
Rotamers (%)	
Outliers	0.1
Favored	95.03
Cβ outliers (%)	0.09
Peptide plane (%)	
Cis proline/general	0/0
Twisted proline/general	0/0

Table S1. Data collection, model composition, refinement, and validation parameters.
